# Evolutionary dynamics of Usutu virus: Worldwide dispersal patterns and transmission dynamics in Europe

**DOI:** 10.3389/fmicb.2023.1145981

**Published:** 2023-03-23

**Authors:** Marina Siljic, Rastko Sehovic, Marko Jankovic, Gorana Stamenkovic, Ana Loncar, Marija Todorovic, Maja Stanojevic, Valentina Cirkovic

**Affiliations:** ^1^Faculty of Medicine, Institute of Microbiology and Immunology, University of Belgrade, Belgrade, Serbia; ^2^Department for Genetic Research, Institute for Biological Research “Sinisa Stankovic”, National Institute of Republic of Serbia, University of Belgrade, Belgrade, Serbia; ^3^Institute for Biocides and Medical Ecology, Belgrade, Serbia; ^4^Group for Medical Entomology, Centre of Excellence for Food- and Vector-Borne Zoonoses, National Institute of Republic of Serbia, Institute for Medical Research, University of Belgrade, Belgrade, Serbia

**Keywords:** Usutu virus, phylogeography, phylodynamics, evolutionary dynamic, mosquito vectors, emerging disease

## Abstract

**Background:**

Usutu virus (USUV) is an emerging mosquito-borne Flavivirus, with birds as the main zoonotic reservoir. Humans are accidental hosts and mostly develop mild or even asymptomatic infections, although severe complications such as encephalitis can also arise. Detailed characterization of the pathogen's phylogenetics may offer valuable insights into the prediction and prevention of potential epidemics; however, lack of uniformity and the number of available USUV sequences worldwide hamper comprehensive investigation.

**Aim:**

The study aimed to investigate USUV spatio-temporal dispersal inter- and intracontinentally and to estimate the dynamics of viral spread within Europe.

**Methods:**

Phylogeographic and phylodynamic analyses were done using advanced phylogenetic methods implemented in Beast 1.10.4 and Beast 2.6.4 software packages.

**Results:**

Herein, we report on a new USUV isolate from *Culex pipiens* collected in 2019 from Serbia. The results of this research revealed two newly described intercontinental migration events of USUV from Africa to Germany in the 1970s and from Africa to the Middle East (Israel) in the late 90s. Finally, phylodynamic analysis substantiated the ongoing active expansion of USUV in Europe.

**Conclusion:**

The data would imply a high potential for further USUV expansion in Europe. Detailed phylogenetic characterization of the pathogen may offer valuable insights into prediction and prevention of potential epidemics; however, lack of uniformity and number of available USUV sequences worldwide hampers comprehensive investigation. This study draws attention to the need for upscaling USUV surveillance.

## Introduction

Arboviruses are among the most important emerging human pathogens, with the threat from mosquito-borne arboviruses extending to livestock and wildlife, with potentially far-reaching consequences for human life and wellbeing (Johnson et al., 2018). Usutu virus (USUV) is an emerging zoonotic agent that may further fuel the arboviral disease burden.

A member of the *Flaviviridae* family, USUV is an arbovirus that was first isolated in South Africa in 1959 (Clé et al., 2019). Its single-stranded positive RNA genome contains a single open reading frame (ORF) coding for a polyprotein of 3,434 amino acids that yields three structural proteins (C—capsid, prM—premembrane prM, and E—envelope), along with eight non-structural proteins (NS1/NS1', NS2a, NS2b, NS3, NS4a, 2K, NS4b, and NS5) (Calisher and Gould, 2003).

Usutu virus is closely related to the more widely known West Nile virus (WNV) and Japanese encephalitis virus (JEV) (Ashraf et al., 2015). The life cycle of the USUV involves mosquitoes of the genus *Culex* as main vectors and various species of birds as amplifying hosts. The virus itself is harmful to birds and known to cause mass extinction events among common blackbirds (*Turdus merula*) and other species of wild birds across Africa and Europe (Clé et al., 2019; Störk et al., 2021). Humans and other mammals are considered incidental hosts, with a limited number of human cases reported so far (Vilibic-Cavlek et al., 2020).

Usutu virus isolates were first phylogenetically classified into seven distinct genetic lineages: three African (Africa 1–3) and four European (Europe 1–Europe 4) (Calzolari et al., 2012; Vilibic-Cavlek et al., 2020). In recent years, with an increasing number of USUV sequences available in databases, one additional European lineage (Europe 5), including sequences from Germany, has also been proposed (Cadar et al., 2017a). Upon its discovery near the Usutu River in Swaziland, South Africa, in 1959, USUV was initially considered endemic to Africa. The first USUV emergence on European soil was noted in 1996 in Italy, based on the retrospective analysis of archived tissue samples originating from a bird die-off (Cadar et al., 2017a).

A massive outbreak in 2001 among common blackbirds in Vienna, Austria, prompted more vigilant surveillance of this new emerging arbovirus. In the following years, USUV has been detected by PCR in mosquitoes in numerous European countries (Germany, Italy, Czech Republic, Croatia, Serbia, Spain, Belgium, and France) as well as serologically in birds throughout Europe (Jöst et al., 2011; Calzolari et al., 2012, 2017; Klobucar et al., 2016; Cadar et al., 2017a; Camp et al., 2019; Clé et al., 2019; Hönig et al., 2019; Vilibic-Cavlek et al., 2020). Since different mammal species serve as incidental hosts of USUV, antibodies have sporadically been detected in horses (Barbic et al., 2013; Csank et al., 2018), dogs (Montagnaro et al., 2019), squirrels (Romeo et al., 2018), wild boar, roe deer (Bournez et al., 2019), and lizards (Csank et al., 2019). Furthermore, USUV RNA has also been found in bats (*Pipistrellus pipistrellus*) in Germany and Belgium (Cadar et al., 2014; Benzarti et al., 2020).

The first two cases of human infection in Africa were described in 1981 and 2004, with both patients presenting with mild symptoms including fever, jaundice, and rash (Nikolay et al., 2011). Cases of more severe disease were described in Italy in 2009 when immunosuppressed patients presented with USUV-associated meningoencephalitis (Cavrini et al., 2009; Pecorari et al., 2009). Subsequently, the only USUV neuroinvasive infection in humans outside Italy was described in Croatia in 2013 during a WNV outbreak (Santini et al., 2015). In 2018, USUV spread rapidly in Western Europe, concomitant to a large WNV outbreak that reached 1,503 human WNV cases, including 181 deaths in a dozen European countries (Clé et al., 2019). In recent years, over 40 symptomatic patients have been reported, as well as several asymptomatic infections fortuitously discovered mostly through routine screening for WNV (Cadar et al., 2017b; Carletti et al., 2018; Cadar and Simonin, 2022).

To date, studies tracing the epidemic origin, USUV inter- and intra-continental migration patterns, and phylodynamics are very scarce. With this study, we aimed to explore USUV spatio-temporal dispersal outside of Africa, and further estimate the dynamics of viral spread within Europe.

## Materials and methods

### Phylogenetic investigation

The present study included USUV NS5 gene sequences, available in the GenBank NCBI database (accessed March 2022), with known location and collection date (Supplementary Table S1). The dataset included one newly generated sequence from Serbia, obtained from a *Culex pipiens* mosquito pool collected in 2019 (more detailed data on the methodology and location are provided in the Supplementary material). Sequence alignment was done by MAFFT 7 software (https://mafft.cbrc.jp/alignment/server/) and manually inspected. The best-fitting nucleotide substitution model for the final sequence data set was GTR+G+I, as selected by Akaike's information criterion (AICc) using jModelTest 3.06 (Shapiro et al., 2006; Posada, 2008). Detection of possible recombination was performed using various models implemented in the Recombination Detection Program v4 (RDP4) (Martin et al., 2015). The sequences suggested to be recombinant by at least three methods were removed. The phylogenetic signal was assessed using likelihood-mapping, which generates maximum-likelihood (ML) trees for all possible quartets of sequences and counts the frequency of trees with varying quality (Strimmer and von Haeseler, 1997). Likelihood-mapping analysis was conducted using the appropriate sets of parameters, including the best-fitting model and 10,000 randomly selected quartets (groups of four randomly chosen sequences). In order to determine the extent to which the data included the temporal structure and to estimate the rate and time scale of USUV evolution, we employed the TempEst program (Rambaut et al., 2016). The input data for this program is a phylogenetic tree that was previously created using the maximum-likelihood (ML) approach using MEGA X software (Kumar et al., 2018). The obtained data were visualized as a distribution chart of root-to-tip distances (a regression against sampling date for dated tips). In order to investigate mutations related to intra- and inter-continental migration events, we analyzed the alignment of amino acid sequences manually and further by employing visual signature pattern analysis software (VisSPA) V 1.6 (https://sray.med.som.jhmi.edu/SCRoftware/VisSPA/).

### Phylogeographic analysis

To describe the geographic dispersal of USUV, phylogeographic analysis was done in the BEAST 1.10.4 software package (https://beast.community/) using the continuous-time Markov Chain (CTMC) process over discrete sampling locations, together with the Bayesian stochastic search variable (BSSV) algorithm (Lemey et al., 2009; Suchard et al., 2018). The uncorrelated lognormal relaxed clock model (UCLN) was chosen as the best fitting, according to the number of previously reported studies, which focused on the in-depth phylogenetic analysis of USUV and West Nile virus (Engel et al., 2016; Fall et al., 2017; Tomazatos et al., 2019; Zecchin et al., 2021). We employed a GMRF Bayesian *Skyride* as a non-parametric coalescent model, in order to take a flexible approach to demographic modeling; this model is used to capture complex population dynamics and MCMC chains were run for 5 × 10^7^ generations for each data set, with a burn-in of 10%. The convergence of parameters was assessed through the ESS>200 after excluding an initial 10% for each run, checked using Tracer v1.6 (Rambaut et al., 2012). The location annotated maximal clade credibility (MCC) tree was visualized using FigTree software v 1.4.4 and analyzed further in the SPREAD3 program. Routes with a posterior probability of >0.6 were considered significant (Rambaut, 2009; Bielejec et al., 2016).

### Phylodynamic investigation (effective reproductive number estimation)

Investigation of the phylodynamics of USUV dispersal in Europe was performed for all distinctly defined phylogenetic clades spreading in Europe consisting of nine or more sequences. Phylodynamic analyses were performed in the BEAST2 v 2.6.5 software package (http://www.beast2.org/) using literature-informed sets of parameters (Stadler et al., 2013; Bouckaert et al., 2014, 2019; Veo et al., 2019). Briefly, the Birth-Death Skyline Serial model (BDSKY) was selected as mosquitoes were sampled sequentially through time and the value of Re was set as a log-normal prior, with a mean value (M) of 0.0 and a variance (S) of 1.25, with the number of dimensions set to 4, 5 or 10 dimensions, as best suited for the particular clade. The rate of becoming uninfectious was set as a normal prior with M = 27 and S = 5 (95% confidence interval 18.8–35.2, corresponding to an infectious period of between 10.4 and 19.4 days), as reported previously (Veo et al., 2019) A prior beta (1.0, 9999) was used to estimate the sampling probability, which corresponded to a minority of cases sampled. The epidemic origin was estimated using a log normal prior with M = 3.0 and S = 0.2. To visualize Re trends, the log output files of BEAST 2 were plotted using the “bdskytools” package in the R studio, available on GitHub. (https://github.com/cran).

## Results

### Phylogenetic analysis

**Figure 1:**
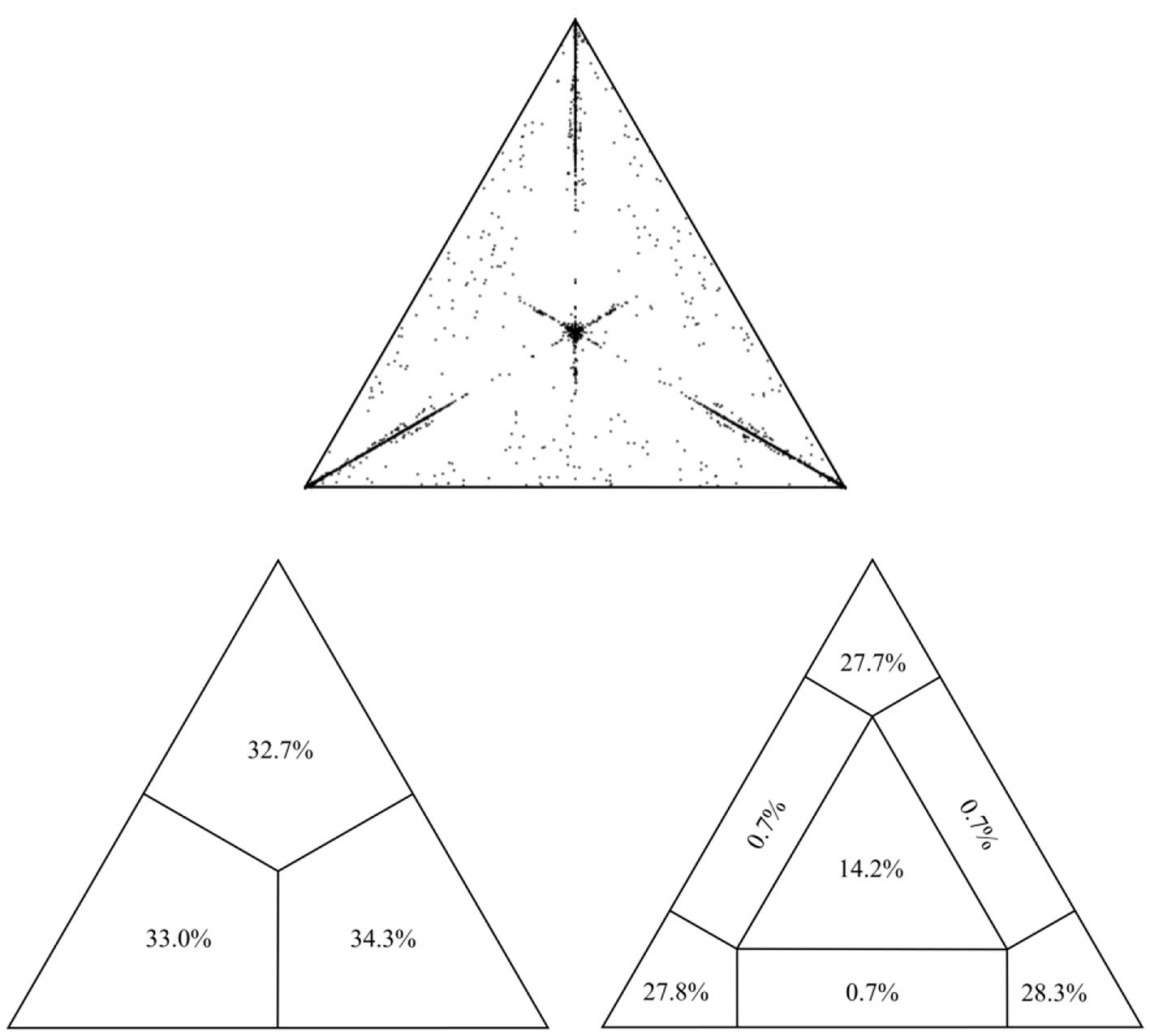
Assessment of the phylogenetic noise of the dataset through the likelihood mapping method implemented in the program TreePuzzle, suggesting sufficient phylogenetic information within the alignment.

The final alignment for the present study encompassed 493 sequences of the USUV partial *NS5* gene (864 nt in length) collected between 1959 and 2020 from 19 countries across Europe, Africa, and Asia (Supplementary Table S1). All sequences were confirmed as non-recombinant by various methods for recombination detection implemented in RDP4. The assessment of phylogenetic noise of the studied USUV *NS5* region, through investigation of 10,000 randomly chosen quartets by means of likelihood mapping, showed that only 16.3% fell in the central area of the likelihood map. The remaining 83.7% were at the corners of the triangle which implies sufficient phylogenetic information (Figure 1). Root-to-tip regression analysis, with an obtained correlation coefficient of 0.37, indicated the sufficient temporal structure of the examined data set, appropriate for in-depth phylogenetic analyses (Supplementary Figure S2).

First, analysis was performed with the aim to compare both isolates from Serbia to the reference sequence by means of BLAST analyses and computation of pairwise distance in MEGA software. Thus far, there is a single reported reference sequence of USUV, isolated from birds, in Austria, with the accession number NC00651. Both the BLAST tool and MEGA software revealed congruent results, revealing the genetic identity of 97.95% and 99.5% of newly detected and previously reported sequences, respectively, compared to the reference sequence. Observed similarity-based amino acid sequences revealed 1.8% and 0.3% of newly detected and previously reported sequences, respectively, compared to the reference sequence.

### Phylogeographic analysis

**Figure 2:**
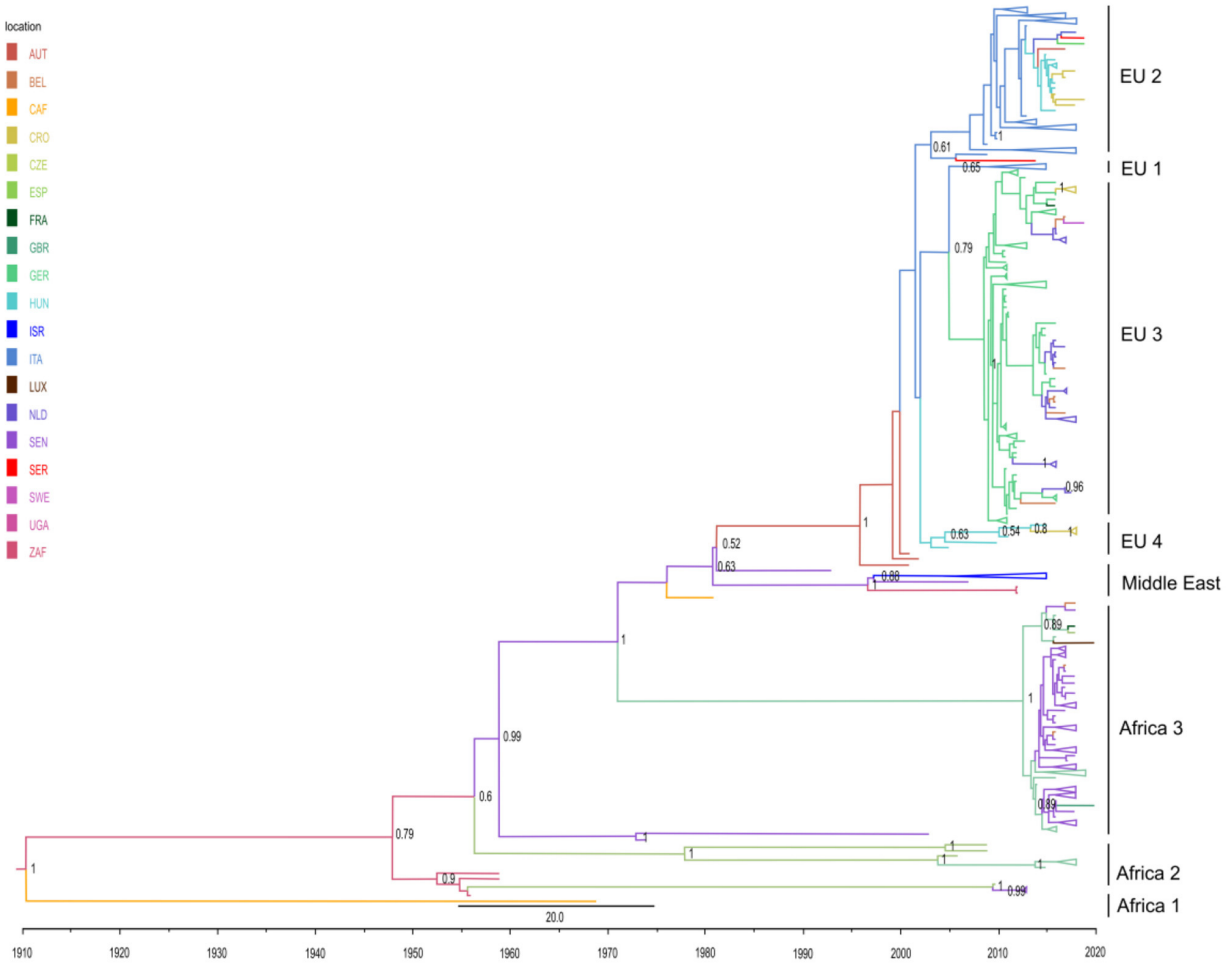
Phylogeographic analysis of 493 partial *NS5* USUV sequences performed in BEAST 1.10.4 software. Maximum clade credibility (MCC) tree was visualized in FigTree 1.4.4. The branches are colored based on the most probable location of the descendent nodes. The numbers on the internal nodes indicate significant posterior probabilities (pp > 0.5), and the scale at the bottom of the tree represents calendar years. All clades with a number of sequences higher than 5 were comprised. Abbreviations: AUT (Austria), BEL (Belgium), CAF (Central African Republic), CRO (Croatia), CZE (Czech Republic), ESP (Spain), FRA (France), GBR (Great Britain), GER (Germany), HUN (Hungary), ISR (Israel), ITA (Italy), LUX (Luxembourg), NLD (The Netherlands), SEN (Senegal), SER (Serbia), SWE (Sweden), UGA (Uganda), ZAF (South Africa).

Herein, a discrete trait phylogeographic analysis was used in order to analyze the viral migration routes and to explore the origin of the USUV outbreaks, with a special focus on viral spread outside Africa. The topology of the obtained MCC tree was in line with the seven previously described phylogenetic lineages: three African (Africa 1–3) and four European (Europe 1–Europe 4) (Figure 2). In addition, we characterized one putative new lineage—the Middle East—consisting of sequences from Israel, Senegal, and Uganda.

The obtained phylogeographic estimates imply that USUV first originated in South Africa at the beginning of the 20th century, and initially spread in Africa (Senegal and Central African Republic). A single highly divergent isolate from the Central African Republic (GenBank accession no. KC754958) thus forms a lineage Africa 1 (Figure 2; Supplementary Figure S3). Subsequently, several intercontinental viral migration events occurred, starting from the mid-20th century [results supported by high posterior probability (pp >0.6)]. Of the estimated five independent intercontinental migration events, four are from Africa to Europe and one from Africa to the Middle East. In detail, two initial intercontinental migration events from Africa (Senegal) to Western Europe (Spain) occurred in the mid-1950's. Upon second entry to Spain around 1956 (95% high-probability density [HPD] from 1930 to 1966), USUV continued to spread intra-continentally to Germany almost 50 years later and further on to France (lineage Africa 2) (Figure 2).

Furthermore, two ensuing migration events in Europe have been estimated: in 1971 in Germany (corresponding to previously designated lineage Africa 3) (95% high-probability density [HPD] for 1958 to 1978) and in 1981 in Austria (95% high-probability density [HPD] for 1969 to 1991). The former clade, representing a newly described entry from Africa (Senegal) to Germany, predominantly contains isolates from the Netherlands and Germany, with sequences from Belgium, France, Luxembourg, Great Britain, and the Czech Republic also taking part. Regarding the latter clade, upon its ingress to Austria, USUV continued to spread throughout West and Central-Eastern Europe *via* several intra-continental migration events between Italy, Hungary, the Czech Republic, Croatia, Serbia, Germany, Austria, Belgium, France, Sweden, and the Netherlands, thus forming a large monophyletic clade (Figure 2) that gave rise to four separate subclades (Europe 1–Europe 4), all except Europe 1 supported by pp > 0.6. The subclade Europe 1 encompasses sequences from Hungary and the Czech Republic. Subclade Europe 2 is the largest one (177 sequences), predominantly of isolates from Italy, which spread from Austria in 2003 (95% high-probability density [HPD] from 1997 to 2004). This subclade contains a distinct heterogeneous Central European cluster consisting of strains from Italy, Hungary, Serbia, Croatia, the Czech Republic, and Germany. Another large subclade, Europe 3 (138 sequences) formed by USUV spread from Italy to Germany in 2005 (95% high-probability density [HPD] from 2005 to 2009), with the majority of sequences from Germany, but also from Belgium, France, Czech Republic, and Sweden. The fourth subclade, Europe 4, is a small cluster of nine Italian sequences.

Finally, our phylogeographic analysis, for the first time, revealed an intercontinental migration of USUV from Senegal to the Middle East (Israel), estimated to have happened in 1997 (95% high-probability density [HPD] from 1995 to 2003). This clade would represent a new putative phylogenetic lineage in the Middle East.

Investigated alignment of the NS5 genetic region (encompassing from 505 to 792 amino acid position) did not reveal any geographically related point mutation.

### Phylodynamic investigation

**Figure 3:**
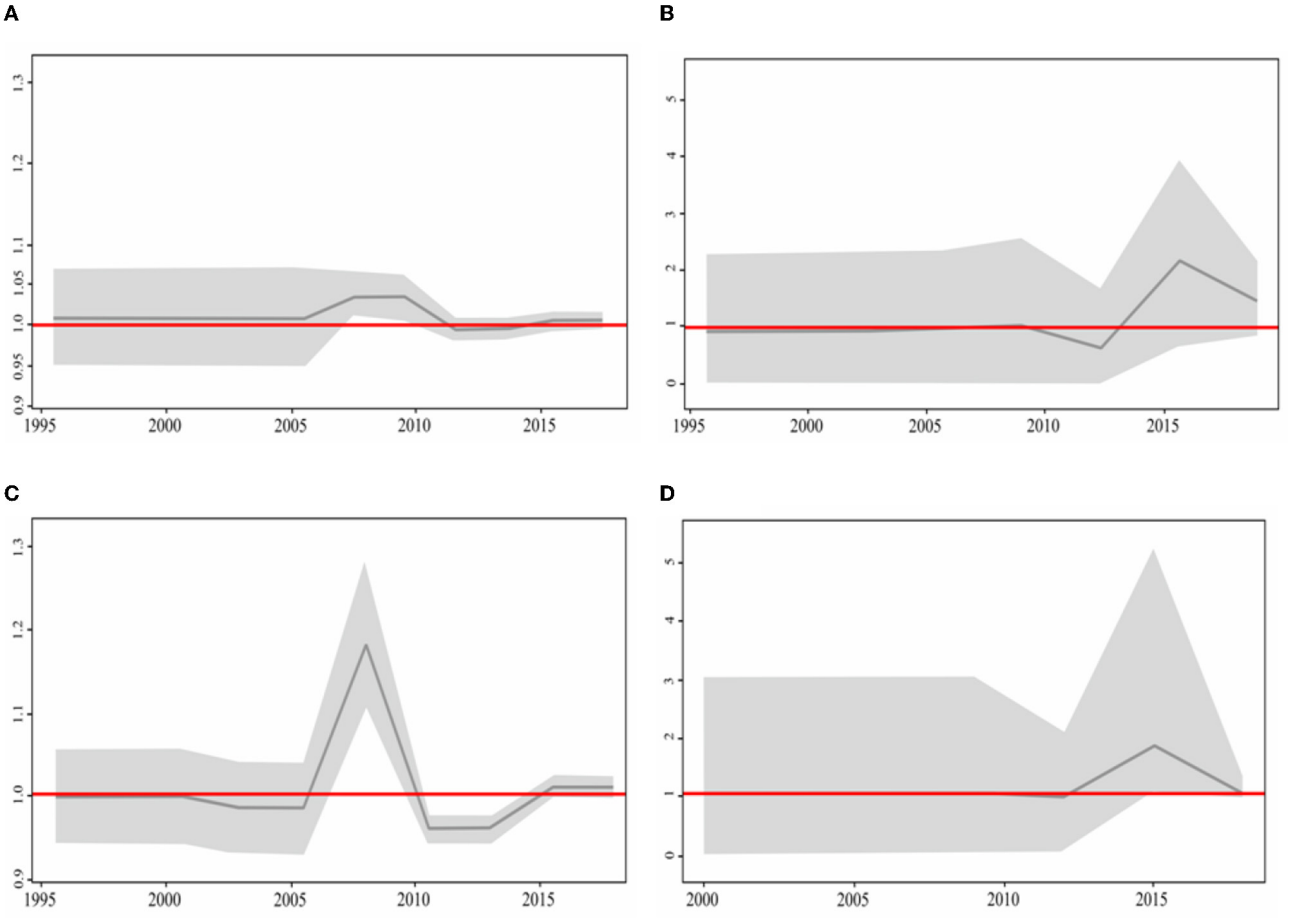
Birth–death skyline plot based on the **(A)** Italian dataset together with the subclade of Central European sequences, **(B)** Central European dataset, **(C)** Europa 3, and **(D)** Africa 3. The BDSKY model was used and implemented in BEAST2 v2.6.5. The red line delineates the cut-off value of Re=1. The shaded area represents 95% confidence intervals of Re estimates over time. The x-axis represents time in years and the y-axis the Re value.

Usutu virus phylodynamics in Europe were assessed by calculating the effective reproductive number (Re) over time on the four total phylogenetic clades: the clade Europe 2, containing predominantly Italian sequences (encompassing 177 sequences in total); its intermixing subclade of 16 sequences from Central-Eastern Europe, including the newly generated one from Serbia; the clade Europe 3, predominantly made of sequences from Germany (encompassing 138 sequences); and the clade Africa 3 (stemming from the newly described entry event to Europe *via* Germany), that mostly contained sequences from the Netherlands (encompassing 100 sequences) (Figures 3A–D).

Re of the clade containing all Italian sequences reached a maximum value in 2009, followed by a decreasing phase and a subsequent rise around 2016 (Figure 3A). Re of the Netherlands cluster reached a maximum in 2015 (Figure 3B). The German sequence started to spread in 2005 and 2 years later reached the maximum (2007). Soon after, it decreased and became inactive until 2015 (Figure 3C). The Central-Eastern Europe dataset started to be active in 2012 and reached its maximum in 2015 (Figure 3D). For all investigated clades, the Re value remained above one until the present time. In particular, for the clades Africa 3 and Central European subclade within the clade Europe 2, Re showed a sharp increase reaching a value of ~2, suggesting the high potential for further expansion.

## Discussion

The present study explores USUV worldwide dispersal patterns and transmission dynamics, with a particular focus on viral spread within Europe, by employing phylogeographic and phylodynamic analysis. With 493 partial USUV *NS5* sequences included, from 19 countries and three continents, this study is the most comprehensive USUV phylogenetic study to date on the number of isolates and their geographic width. This study is, to the best of our knowledge, only the second to explore USUV phylogeographic spread between continents, and the first to present USUV phylodynamics in Europe, by employing the Bayesian phylogenetic approach. The *NS5* gene was chosen as the one with the greatest nucleotide and amino acids variation among all commonly analyzed USUV genes (Engel et al., 2016). Of note, phylogenetic studies based on the NS5 gene sequences have been shown to correlate well with the existing USUV whole genome data (Engel et al., 2016; Zecchin et al., 2021).

The expanded scope and range of arboviral infections lie among the issues that arise from climate change, impinging on many facets of life, and human health and wellbeing are no exception (Grubaugh and Ebel, 2016; Grubaugh et al., 2017). Extended local and global dispersion of arboviruses is a driver of pathogen evolution, as viruses adapt to local ecological conditions (Júnior and Mendonça, 2021). This may result in higher human morbidity and mortality. Comprehensive phylogenetic research, such as our study, coupled with vector surveillance may reinforce disease prevention.

Several studies to date have explored USUV genetic diversity (Auguste et al., 2010; Cadar et al., 2015; Engel et al., 2016; Ziegler et al., 2016; Zecchin et al., 2021). Nonetheless, the phylogenetic classification of USUV has not been completely elucidated yet, with a different number of described lineages, in relation to the size of the studied dataset (in sequence number and length). We explored USUV phylogeographic migration patterns, and the results obtained are mostly in line with previous studies that classified USUV into seven phylogenetic lineages, corresponding to independent migration paths in our analysis. Of note, a novel migration event from Senegal to Israel and a phylogenetic clade including isolates from this country have been described in our study, and tentatively classified as Middle East lineage. Overall, we observed eight distinct phylogeographic lineages: three African, four European, and one new Middle East clade. These lineages stem from five intercontinental migration events out of Africa: four toward Europe and one toward the Middle East.

The obtained results imply that the initial USUV spread at the beginning of the 20th century was fairly local throughout Africa (South Africa, Senegal, and Central African Republic); however, the existing sequence data do not allow for reliable and accurate reconstruction of the initial USUV African intra-continental dispersal. The initial point of entry to Europe has been estimated to be Spain in the mid-1950's, on at least two separate occasions, both originating from Senegal and forming the genetic lineage Africa 2. Similar findings have been described by Engel et al., however, on a smaller dataset including full genome sequences and partial *E* and *NS5* gene sequences (Engel et al., 2016). Even though the virus entered Spain in the mid-1950's, it remained restrained locally, with the further intracontinental spread of this lineage in Europe starting at the beginning of the 2000's. A similar pattern of delayed dispersal until the early 2000's is also evident for both the clade Africa 3, formed by the third introduction of USUV from Africa to Europe (Germany), estimated to have occurred in 1971, and the fourth clade, estimated to have occurred in 1981, in Austria.

USUV introduction to Austria further on led to dispersal throughout West and Central – Eastern Europe via a number of intra-continental migration events and thus forming one large monophyletic clade as also shown by Engel et al. (2016). However, the direction of viral spread can be considered as controversial. Several previous studies together with the results obtained in our study suggested the direction of USUV dispersal occurred from Austria to Italy and further on throughout West and Central – Eastern Europe (Engel et al., 2016; Calzolari et al., 2017; Roesch et al., 2019). In the contrast to these findings, Zecchin et al. (2021) proposed that USUV spread from Italy to Austria in two different occasions. The first introduction occurred in the mid nineties, while the second was in 2012. Nevertheless, this result may be the consequence due to a bias of a large number of Italian sequences included in the study. All aforementioned studies have certainly suggested that Italy acted mainly as donor of USUV to neighboring countries, indicating the central role of this country to USUV spread throughout Europe (Zecchin et al., 2021).

The fifth intercontinental route of USUV spread, described for the first time in this study, represented the entry of the virus from Africa to Israel, estimated to have occurred in 1997. Israeli strains share the most recent common ancestor with Senegalese strains. In view of targeted prevention and response to potential outbreaks, further dispersal of this lineage should be carefully monitored, especially considering recent results on the seroprevalence of WNV and USUV detected in horses in Israel. Seroprevalence for neutralizing antibodies against WNV and USUV was 84.1% and 10.8%, respectively, in 2018 (Mannasse et al., 2017; Schvartz et al., 2020).

The results of our phylogeographic analysis suggested that the evolution of USUV has been shaped by long-distance migration routes between continents. Migratory birds are known as viral hosts and reservoirs, hence viral dispersion, including arboviruses such as WNV and USUV, has been associated with migratory birds' flyways (Zhang et al., 2014; Mancuso et al., 2022). Eight major migratory birds' flyways have been established: Pacific Americas, Mississippi Americas, Atlantic Americas, East Atlantic, Black Sea-Mediterranean, West Asia-East Africa, Central Asia, and East Asia/Australasia (CMS Technical Series No. 27, 2014). A link has already been proposed between USUV dispersal from Senegal to Spain and a large East Atlantic intercontinental migratory bird flyway, which encompasses practically the whole of Spain (Engel et al., 2016). The Black Sea-Mediterranean flyway, linking Africa to Central-Eastern Europe could, therefore, be proposed as the main route for the spread of USUV from Africa to Austria and Italy. Considering that these two migration routes overlap on the territory of Western Europe, USUV's introduction to Germany and further into the Netherlands might be explained by either of the two. Similarly, the spread of USUV from Africa to Israel might be connected to bird migration *via* the Black Sea-Mediterranean or West Asia/East Africa migration routes, which have been established for many bird species that breed in the mid-Palaearctic and choose to embark on a much longer south-westerly migration to Africa (Zhang et al., 2014).

In addition to migratory birds' pathways as one of the most important mechanisms for the spread of arboviruses, other ways of USUV dispersion should be taken into consideration. For example, the presence of invasive and exotic mosquito species capable of USUV transmission at large international airports presents a new variable in USUV dissemination routes (Ibáñez-Justicia et al., 2020). Furthermore, regions suitable for *Cx. pipiens* survival, a phenomenon strongly connected to climate change, present the risk of USUV introduction into formerly inaccessible biomes, representing potential epidemiological hotspots (Hongoh et al., 2012).

Previously, Engel et al. postulated that the majority of European USUV strains resulted from a single introduction; however, we have identified two major migration events that led to wider USUV dispersal in Europe: to Germany in 1971 (clades Africa 3) and to Austria in 1981 (a clade further spread through lineages Europe 1–4). The simultaneous presence of USUV lineages Europe 3 and Africa 3 has been noted in Germany and the Netherlands predominantly from blackbirds, raising the issue of enzootic viral co-circulation (Oude Munnink et al., 2020).

In general, in spite of the identified prior introduction events, the substantial spread of different USUV lineages within Europe only started in the early 2000's, with a “lag” period of several decades. If these findings indeed reflect delayed dispersal upon a period of silent, low-scale USUV circulation, this still remains to be elucidated along with the specific circumstances that triggered the spread. To investigate the temporal trend of the USUV epidemic in Europe, we analyzed the phylodynamics of the four largest clades spreading on the continent (clades Europe 2 with its Central European subclade, Europe 3, and Africa 3) predominantly including sequences from Italy (encompassing one Central-Eastern European clade), Germany, and the Netherlands, using a birth-death skyline model. Estimation of the Re value may indicate whether the numbers of pathogen carriers or infected units (IUs) will increase or decrease, with values of Re above 1 suggesting an expansion of the infected population (Achaiah and Subbarajasetty, 2020). For all the analyzed USUV clades, an increase in activity was noted after the 2000's, indicating that USUV intensively spread throughout Europe rather recently. Most zoonotic pathogens are not highly transmissible within human populations and cannot cause major epidemics (Woolhouse and Gowtage-Sequeria, 2005). Consequently, for pathogens that are minimally transmissible within human populations, outbreak size is determined largely by the number of introductions from the reservoir. The Italian epidemic caused by WNV, also a member of the *Flaviviridae* family, was analyzed in the same manner using in-depth phylogenetic analysis. As a result, parallel trends in epidemic growth for the period from 2011–2018 in Italy have been seen for both USUV and WNV (Veo et al., 2019; Zecchin et al., 2021). The fact that both viruses formed endemic clades strengthens the hypothesis of local over-wintering in Italy more than that of the annual reintroduction of the same viral strain (Veo et al., 2019).

To a certain extent, a limiting factor to our study may be the lack of USUV WGS analysis. Nevertheless, there is evidence of WGS and sole-*NS5* analyses yielding very congruent results regarding topologies and introduction events inferred by maximum likelihood (ML) and Bayesian maximum clade credibility (MCC) phylogenies. An example is present in the study of Engel et al. (2016), where WGS and *NS5* Sanger sequencing yielded similar results. Furthermore, we used the largest number of *NS5* sequences for the phylogenetic analysis so far (493, as opposed to 406 sequences available in the NCBI database), hopefully giving a clear and informative representation of the phylogenetic properties of USUV's temporal and geographical spread. Finally, the *NS5* gene was chosen for its greatest nucleotide and amino acid variation among all commonly analyzed USUV genes (Engel et al., 2016). Finally, we would be remiss not to mention as a possible drawback a discrepancy in the accuracy of the estimated global times to the most recent common ancestor (TMRCA) for the *NS5* and complete genome data sets. However, the estimated TMRCAs for each lineage (except Africa 1) were very similar in the *NS5* and complete genome MCC trees. The same results were obtained herein. In addition, the existence of eight different USUV lineages has been proposed by Cadar et al. (2017a). In their study, the authors analyzed USUV sequences of different lengths. Therein, those of the newly identified lineage Europe 5 (KY113091, KY113097, and KY113101–KY1130104) were 265 nt in length, none of which overlapped with our alignment. Therefore, it was not possible to include these sequences in the present study; another reason for not including the sequences from Germany is the lack of WGS analysis of the available USUV genomes in our research. Admittedly, this may also present a potential limitation of our investigation.

The present study represents the most comprehensive in-depth phylogenetic analysis of USUV, based on 493 isolates originating from three continents. By using the most comprehensive USUV sequence dataset so far, we have identified two new virus introduction events in Europe and the Middle East. Estimated Re values for the dominant clades spreading in Europe imply the high potential for further expansion. Detailed phylogenetic characterization may offer valuable insights into the prediction and prevention of potential epidemics; however, the lack of uniformity and the number of available USUV sequences worldwide hamper comprehensive investigation.

## Data availability statement

The datasets presented in this study can be found in online repositories. The names of the repository/repositories and accession number(s) can be found in the article/Supplementary material.

## Author contributions

MJ, VC, and MSi conceived and designed the study and wrote the manuscript. VC, MSi, RS, MT, and AL performed the analyses. MSt and GS supervised the study, reviewed, and critically edited the manuscript. All authors have read and agreed to the submitted version of the manuscript.
