# Supplementary material for: Evolutionary dynamics of Usutu virus: Worldwide dispersal patterns and transmission dynamics in Europe
Source: Front Microbiol. 2023 Mar 23;14:1145981. doi: 10.3389/fmicb.2023.1145981 (PMC10076808; doi:10.3389/fmicb.2023.1145981)
Supplement: Supplementary file 1 [file Data_Sheet_1.doc]

**Conventional PCR and sequencing**

Mosquitoes were collected within West Nile virus (WNV) surveillance program in Belgrade and surroundings, which is carried out by the Institute for Biocides and Medical Ecology in Belgrade, Serbia. Within this program, one USUV positive mosquito pool was detected in the city of Sabac in 2019 (Figure 1). In order to genetic characterize the newly detected isolate from Serbia and to compare it with other sequences available in GenBank, this RNA was further on analyzed using conventional molecular methods and in-depth phylogenetic analysis within the project entitled “*An evolutionary insight into molecular diversity of emerging pathogens in Serbia through phylogenetic approach*” (https://indepth.rs/en/), at the Institute of Microbiology and Immunology, Faculty of Medicine. For amplification of NS5 gene of USUV, one step RT PCR was initiated using 3 μl of RNA, 20 pmol of, sense 1NS5F-GCATCTAYAWCAYNATGGG and antisense 1NS5R-CCANACNYNRTTCCANAC primers, buffer and PrimeScript Enzyme Mix in a final volume of 25 μl (TaKaRa Shuzo Co., LTD, Shiga, Japan). Second round of PCR (50 μl), that was conducted using DreamTaq Hot Start PCR Master Mix (Thermo Fisher Scientific, Massachusetts, USA**)** contained 5 μl DNA from the first-round reaction, 20 pmol each of sense 2NS5F-GCNATNTGGTWYATGTGG and antisense 2NS5R-TRTCTTCNGTNGTCATCC primers. Both rounds of nested PCR included degenerate primers that were previously constructed and described for the Ns5 gene of WNV (34). Amplified product was directly sequenced following purification using the GeneJet Purification Kit (Thermo Fisher Scientific, (Thermo Fisher Scientific, Massachusetts, USA). Sequencing was performed using second round forward and reverse primers and the BigDye Version 3.0 Dye Terminator Cycle Sequencing Kit (Applied Biosystems, Foster City, CA) on an ABI 3730 automated capillary sequencer. Sequence was visually inspec ted, edited manually and assembled into a single contig.

Within the standard WNV environmental screening program carried out by the Institute for Biocides and Medical Ecology in Belgrade, USUV RNA was detected in one mosquito pool. Insects within this pool were collected in the city of Sabac in 2019 (Figure S1) and morphologically characterized as *Culex pipiens*. Obtained partial *NS5* gene sequence of USUV (1014 nt in length) was deposited in GenBank under the accession number MW803140. BLAST analysis of the obtained nucleotide sequence indicated the highest similarity to sequences isolated from birds in the Czech Republic, Germany and Austria (Accession no. MN419913, LR989890 and MF063042) with 97.54%.

**
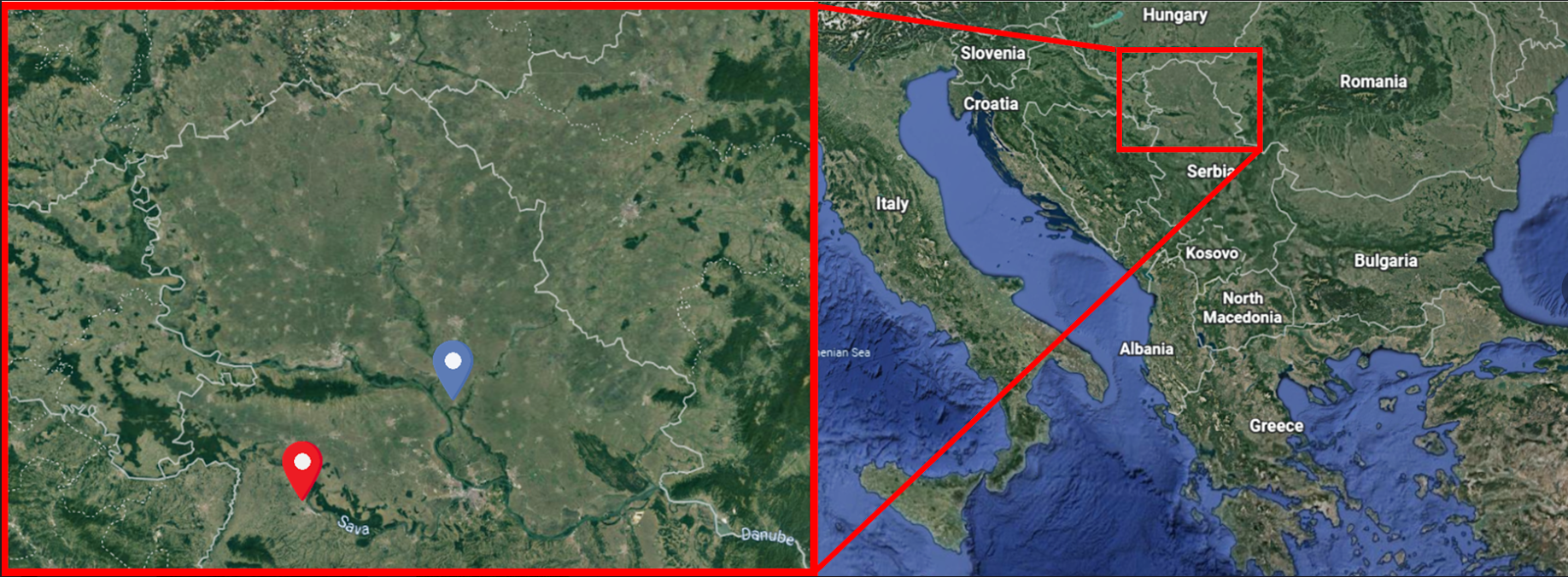
**

**Figure S1.** Map of the north of Serbia (left) with reference to its overall position in Southeast Europe (right). The red pin indicates the latest, novel site of detection of USUV (this work), while the blue pin marks the mosquito-sampling location where the virus was detected previously.


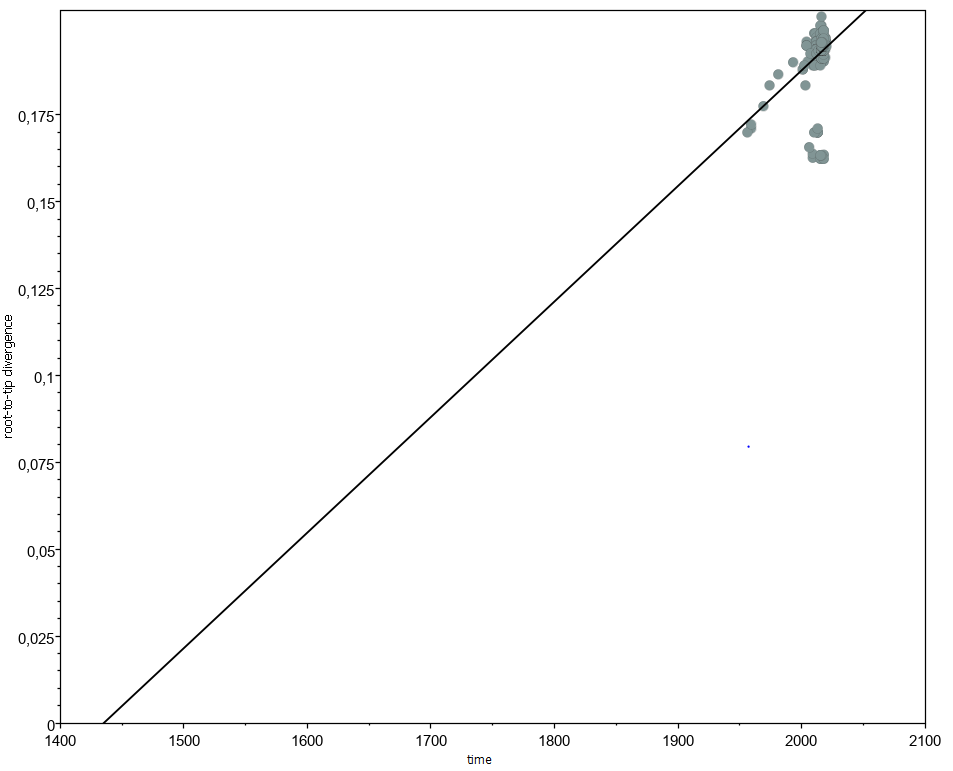


**Figure S2**. Root-to-tip analyses obtained with TempEst, using ML reconstruction obtained from 493 USUV sequences; graph correlation between time (years) and genetic divergence (substitutions per site) from the root of the tree to the tips (sampled genomes) is shown.


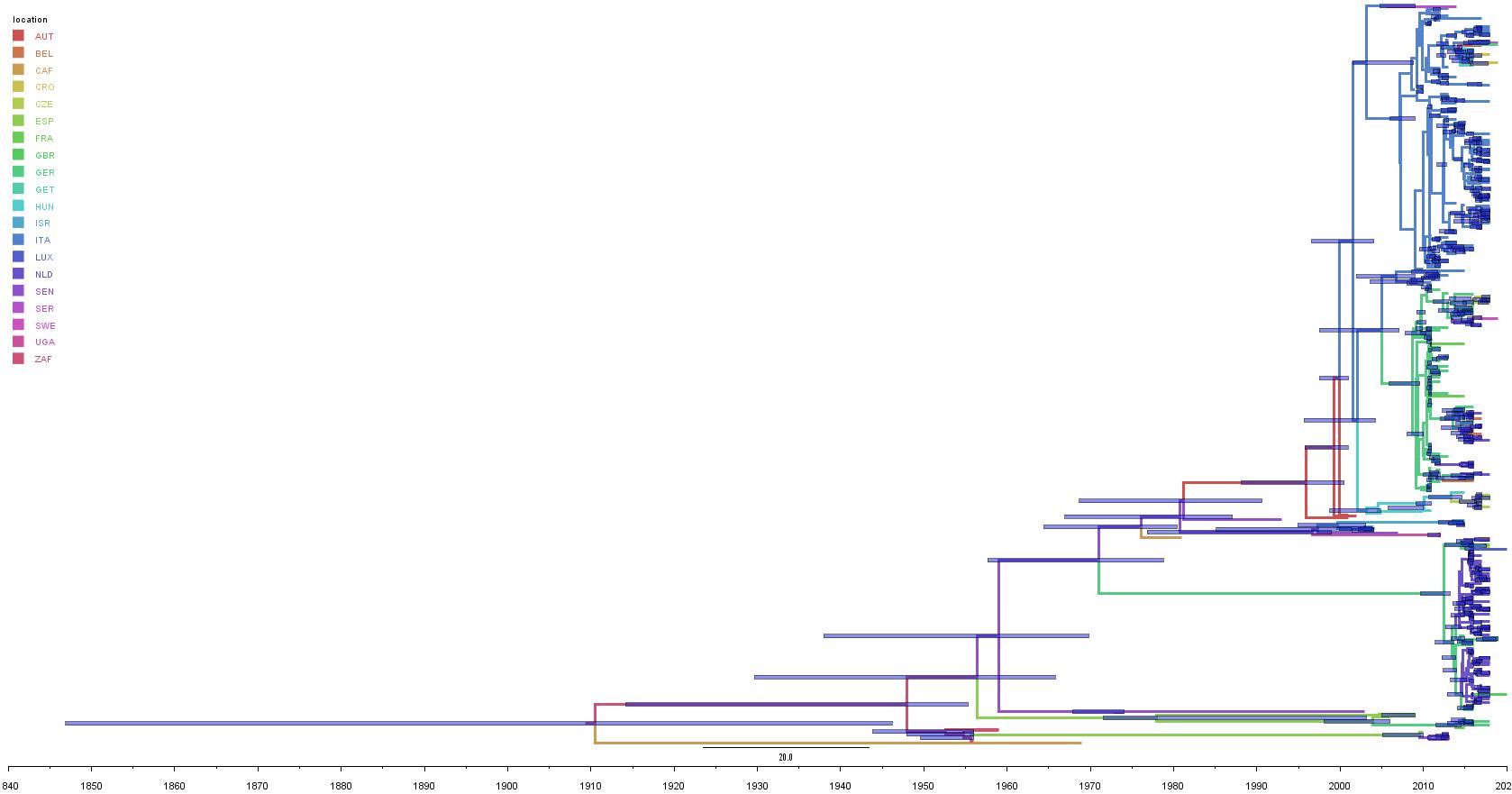


Figure S3. Phylogeographic analysis of 493 partial NS5 USUV sequences performed in BEAST 1.10.4 software. Maximum clade credibility (MCC) tree was visualized in FigTree 1.4.4. The branches are colored based on the most probable location of the descendent nodes. The numbers on the internal nodes indicate significant posterior probabilities (pp > 0.5), and the scale at the bottom of the tree represents calendar years. Error bars represent the 95% highest posterior density (HPD)Abbreviations: AUT (Austria), BEL (Belgium), CAF (Central African Republic), CRO (Croatia), CZE (Czech Republic), ESP (Spain), FRA (France), GBR (Great Britain), GER (Germany), HUN (Hungary), ISR (Israel), ITA (Italy), LUX (Luxembourg), NLD (The Netherlands), SEN (Senegal), SER (Serbia), SWE (Sweden), UGA (Uganda), ZAF (South Africa).

| Acc number | Location | Year | Host/Vector |
| --- | --- | --- | --- |
| MT784898 | Italy | 2017 | Bird |
| MT784899 | Italy | 2017 | Bird |
| MW164635 | Italy | 2011 | Mosquito |
| MW164636 | Italy | 2011 | Mosquito |
| MW164637 | Italy | 2011 | Mosquito |
| MW164638 | Italy | 2011 | Mosquito |
| MW164639 | Italy | 2011 | Mosquito |
| MW164640 | Italy | 2011 | Mosquito |
| MW164641 | Italy | 2012 | Mosquito |
| MW164642 | Italy | 2012 | Mosquito |
| MW164643 | Italy | 2012 | Mosquito |
| MW164644 | Italy | 2012 | Mosquito |
| MW164645 | Italy | 2012 | Mosquito |
| MW164646 | Italy | 2012 | Mosquito |
| MW164647 | Italy | 2012 | Mosquito |
| MW164648 | Italy | 2012 | Mosquito |
| MW164649 | Italy | 2012 | Mosquito |
| MW164650 | Italy | 2012 | Mosquito |
| MW164651 | Italy | 2012 | Mosquito |
| MW164652 | Italy | 2012 | Mosquito |
| MW164653 | Italy | 2012 | Mosquito |
| MW164654 | Italy | 2012 | Bird |
| MW164655 | Italy | 2012 | Bird |
| MW164656 | Italy | 2012 | Bird |
| MW164657 | Italy | 2012 | Bird |
| MW164658 | Italy | 2013 | Mosquito |
| MW164659 | Italy | 2013 | Mosquito |
| MW164660 | Italy | 2013 | Mosquito |
| MW164661 | Italy | 2013 | Mosquito |
| MW164662 | Italy | 2013 | Mosquito |
| MW164663 | Italy | 2013 | Mosquito |
| MW164664 | Italy | 2013 | Mosquito |
| MW164665 | Italy | 2013 | Mosquito |
| MW164666 | Italy | 2013 | Mosquito |
| MW164667 | Italy | 2013 | Mosquito |
| MW164668 | Italy | 2013 | Mosquito |
| MW164669 | Italy | 2013 | Mosquito |
| MW164670 | Italy | 2013 | Mosquito |
| MW164671 | Italy | 2014 | Mosquito |
| MW164672 | Italy | 2014 | Mosquito |
| MW164673 | Italy | 2014 | Mosquito |
| MW164674 | Italy | 2014 | Mosquito |
| MW164675 | Italy | 2014 | Mosquito |
| MW164676 | Italy | 2014 | Mosquito |
| MW164677 | Italy | 2014 | Mosquito |
| MW164678 | Italy | 2014 | Mosquito |
| MW164679 | Italy | 2014 | Mosquito |
| MW164680 | Italy | 2014 | Mosquito |
| MW164681 | Italy | 2014 | Mosquito |
| MW164682 | Italy | 2014 | Mosquito |
| MW164683 | Italy | 2014 | Mosquito |
| MW164684 | Italy | 2014 | Bird |
| MW164685 | Italy | 2015 | Mosquito |
| MW164686 | Italy | 2015 | Mosquito |
| MW164687 | Italy | 2015 | Mosquito |
| MW164688 | Italy | 2015 | Mosquito |
| MW164689 | Italy | 2015 | Mosquito |
| MW164690 | Italy | 2015 | Mosquito |
| MW164691 | Italy | 2015 | Mosquito |
| MW164692 | Italy | 2015 | Mosquito |
| MW164693 | Italy | 2015 | Mosquito |
| MW164694 | Italy | 2015 | Bird |
| MW164695 | Italy | 2015 | Bird |
| MW164696 | Italy | 2015 | Bird |
| MW164697 | Italy | 2016 | Mosquito |
| MW164698 | Italy | 2016 | Mosquito |
| MW164699 | Italy | 2016 | Mosquito |
| MW164700 | Italy | 2016 | Mosquito |
| MW164701 | Italy | 2016 | Mosquito |
| MW164702 | Italy | 2016 | Mosquito |
| MW164703 | Italy | 2106 | Mosquito |
| MW164704 | Italy | 2016 | Mosquito |
| MW164705 | Italy | 2016 | Mosquito |
| MW164706 | Italy | 2016 | Mosquito |
| MW164707 | Italy | 2016 | Mosquito |
| MW164708 | Italy | 2017 | Mosquito |
| MW164709 | Italy | 2017 | Mosquito |
| MW164710 | Italy | 2017 | Mosquito |
| MW164711 | Italy | 2017 | Mosquito |
| MW164712 | Italy | 2017 | Mosquito |
| MW164713 | Italy | 2017 | Mosquito |
| MW164714 | Italy | 2017 | Mosquito |
| MW164715 | Italy | 2017 | Mosquito |
| MW164716 | Italy | 2017 | Mosquito |
| MW164717 | Italy | 2017 | Mosquito |
| MW164718 | Italy | 2017 | Mosquito |
| MW164719 | Italy | 2017 | Mosquito |
| MW164720 | Italy | 2017 | Mosquito |
| MW164721 | Italy | 2017 | Mosquito |
| MW164722 | Italy | 2017 | Mosquito |
| MW164723 | Italy | 2017 | Mosquito |
| MW164724 | Italy | 2017 | Mosquito |
| MW164725 | Italy | 2017 | Mosquito |
| MW164726 | Italy | 2017 | Mosquito |
| MW164727 | Italy | 2017 | Bird |
| MW164728 | Italy | 2017 | Bird |
| MW164729 | Italy | 2017 | Bird |
| MW164730 | Italy | 2018 | Mosquito |
| MW164731 | Italy | 2018 | Mosquito |
| MW164732 | Italy | 2018 | Mosquito |
| MW164733 | Italy | 2018 | Mosquito |
| MW164734 | Italy | 2018 | Mosquito |
| MW164735 | Italy | 2018 | Mosquito |
| MW164736 | Italy | 2018 | Mosquito |
| MW164737 | Italy | 2018 | Mosquito |
| MW164738 | Italy | 2018 | Mosquito |
| MW164739 | Italy | 2018 | Mosquito |
| MW164740 | Italy | 2018 | Mosquito |
| MW164741 | Italy | 2018 | Mosquito |
| MW164742 | Italy | 2018 | Mosquito |
| MW164743 | Italy | 2018 | Mosquito |
| MW164744 | Italy | 2018 | Mosquito |
| MW164745 | Italy | 2018 | Mosquito |
| MW164746 | Italy | 2018 | Mosquito |
| MW164747 | Italy | 2018 | Mosquito |
| MW164748 | Italy | 2018 | Mosquito |
| MW164749 | Italy | 2018 | Mosquito |
| MW164750 | Italy | 2018 | Mosquito |
| MW164751 | Italy | 2018 | Mosquito |
| MW164752 | Italy | 2018 | Mosquito |
| MW164753 | Italy | 2018 | Mosquito |
| MW164754 | Italy | 2018 | Mosquito |
| MW164755 | Italy | 2018 | Mosquito |
| MW164756 | Italy | 2018 | Mosquito |
| MW164757 | Italy | 2018 | Mosquito |
| MW164758 | Italy | 2018 | Mosquito |
| MW164759 | Italy | 2018 | Mosquito |
| MW164760 | Italy | 2018 | Mosquito |
| MW164761 | Italy | 2018 | Mosquito |
| MW164762 | Italy | 2018 | Mosquito |
| MW164763 | Italy | 2018 | Mosquito |
| MW164764 | Italy | 2018 | Mosquito |
| MW164765 | Italy | 2018 | Mosquito |
| MW164766 | Italy | 2018 | Mosquito |
| MW164767 | Italy | 2018 | Mosquito |
| MW164768 | Italy | 2018 | Mosquito |
| MW164769 | Italy | 2018 | Mosquito |
| MW164770 | Italy | 2018 | Bird |
| MW164771 | Italy | 2018 | Bird |
| KF801600 | Italy | 2012 | Mosquito |
| KF801596 | Italy | 2012 | Mosquito |
| KF801595 | Italy | 2012 | Mosquito |
| KF801594 | Italy | 2012 | Mosquito |
| KU573076 | Italy | 2014 | Mosquito |
| KU573075 | Italy | 2014 | Mosquito |
| KU573074 | Italy | 2013 | Mosquito |
| KU573073 | Italy | 2013 | Mosquito |
| KU573072 | Italy | 2013 | Mosquito |
| KU573070 | Italy | 2013 | Mosquito |
| KM871201 | Italy | 2011 | Mosquito |
| KX555629 | Italy | 2015 | Bird |
| KX555628 | Italy | 2010 | Bird |
| KX555627 | Italy | 2010 | Bird |
| KX555626 | Italy | 2010 | Bird |
| KX555625 | Italy | 2010 | Bird |
| KX555624 | Italy | 2010 | Bird |
| KX816653 | Italy | 2012 | Mosquito |
| KX816652 | Italy | 2012 | Mosquito |
| KX816651 | Italy | 2012 | Mosquito |
| KX816650 | Italy | 2012 | Mosquito |
| KX816649 | Italy | 2011 | Bird |
| KX816648 | Italy | 2011 | Mosquito |
| KX816647 | Italy | 2011 | Mosquito |
| KX816646 | Italy | 2010 | Bird |
| MN989418 | Italy | 2018 | Bird |
| KF882514 | Italy | 2011 | Mosquito |
| JF266698 | Italy | 2009 | Bird |
| HM569263 | Italy | 2009 | Human |
| MN122256 | Netherlands | 2018 | Bird |
| MN122255 | Netherlands | 2018 | Bird |
| MN122254 | Netherlands | 2018 | Bird |
| MN122253 | Netherlands | 2018 | Bird |
| MN122252 | Netherlands | 2018 | Bird |
| MN122251 | Netherlands | 2018 | Bird |
| MN122250 | Netherlands | 2018 | Bird |
| MN122249 | Netherlands | 2018 | Bird |
| MN122248 | Netherlands | 2018 | Bird |
| MN122247 | Netherlands | 2018 | Bird |
| MN122246 | Netherlands | 2018 | Bird |
| MN122245 | Netherlands | 2018 | Bird |
| MN122244 | Netherlands | 2018 | Bird |
| MN122243 | Netherlands | 2018 | Bird |
| MN122242 | Netherlands | 2018 | Bird |
| MN122241 | Netherlands | 2018 | Bird |
| MN122240 | Netherlands | 2018 | Bird |
| MN122239 | Netherlands | 2018 | Bird |
| MN122238 | Netherlands | 2018 | Bird |
| MN122237 | Netherlands | 2018 | Bird |
| MN122236 | Netherlands | 2018 | Bird |
| MN122235 | Netherlands | 2018 | Bird |
| MN122234 | Netherlands | 2018 | Bird |
| MN122233 | Netherlands | 2018 | Bird |
| MN122232 | Netherlands | 2018 | Bird |
| MN122231 | Netherlands | 2018 | Bird |
| MN122230 | Netherlands | 2018 | Bird |
| MN122229 | Netherlands | 2018 | Bird |
| MN122228 | Netherlands | 2018 | Bird |
| MN122227 | Netherlands | 2018 | Bird |
| MN122226 | Netherlands | 2018 | Bird |
| MN122225 | Netherlands | 2018 | Bird |
| MN122224 | Netherlands | 2018 | Bird |
| MN122223 | Netherlands | 2018 | Bird |
| MN122222 | Netherlands | 2018 | Bird |
| MN122221 | Netherlands | 2018 | Bird |
| MN122220 | Netherlands | 2018 | Bird |
| MN122219 | Netherlands | 2018 | Bird |
| MN122218 | Netherlands | 2018 | Bird |
| MN122217 | Netherlands | 2018 | Bird |
| MN122216 | Netherlands | 2018 | Bird |
| MN122215 | Netherlands | 2017 | Bird |
| MN122214 | Netherlands | 2017 | Bird |
| MN122213 | Netherlands | 2017 | Bird |
| MN122212 | Netherlands | 2017 | Bird |
| MN122211 | Netherlands | 2017 | Bird |
| MN122210 | Netherlands | 2017 | Bird |
| MN122209 | Netherlands | 2017 | Bird |
| MN122208 | Netherlands | 2017 | Bird |
| MN122207 | Netherlands | 2017 | Bird |
| MN122206 | Netherlands | 2017 | Bird |
| MN122205 | Netherlands | 2017 | Bird |
| MN122204 | Netherlands | 2017 | Bird |
| MN122203 | Netherlands | 2017 | Bird |
| MN122202 | Netherlands | 2017 | Bird |
| MN122201 | Netherlands | 2017 | Bird |
| MN122200 | Netherlands | 2017 | Bird |
| MN122199 | Netherlands | 2017 | Bird |
| MN122198 | Netherlands | 2017 | Bird |
| MN122197 | Netherlands | 2017 | Bird |
| MN122196 | Netherlands | 2017 | Bird |
| MN122195 | Netherlands | 2017 | Bird |
| MN122194 | Netherlands | 2017 | Bird |
| MN122193 | Netherlands | 2017 | Bird |
| MN122192 | Netherlands | 2017 | Bird |
| MN122191 | Netherlands | 2017 | Bird |
| MN122190 | Netherlands | 2017 | Bird |
| MN122189 | Netherlands | 2017 | Bird |
| MN122188 | Netherlands | 2017 | Bird |
| MN122187 | Netherlands | 2016 | Bird |
| MN122186 | Netherlands | 2016 | Bird |
| MN122185 | Netherlands | 2016 | Bird |
| MN122184 | Netherlands | 2016 | Bird |
| MN122183 | Netherlands | 2016 | Bird |
| MN122182 | Netherlands | 2016 | Bird |
| MN122181 | Netherlands | 2016 | Bird |
| MN122180 | Netherlands | 2016 | Bird |
| MN122179 | Netherlands | 2016 | Bird |
| MN122178 | Netherlands | 2016 | Bird |
| MN122177 | Netherlands | 2016 | Bird |
| MN122176 | Netherlands | 2016 | Bird |
| MN122175 | Netherlands | 2016 | Bird |
| MN122174 | Netherlands | 2016 | Bird |
| MN122173 | Netherlands | 2016 | Bird |
| MN122172 | Netherlands | 2016 | Bird |
| MN122171 | Netherlands | 2016 | Bird |
| MN122170 | Netherlands | 2016 | Bird |
| MN122169 | Netherlands | 2016 | Bird |
| MN122168 | Netherlands | 2016 | Bird |
| MN122167 | Netherlands | 2016 | Bird |
| MN122166 | Netherlands | 2016 | Bird |
| MN122165 | Netherlands | 2016 | Bird |
| MN122164 | Netherlands | 2016 | Bird |
| MN122163 | Netherlands | 2016 | Bird |
| MN122162 | Netherlands | 2016 | Bird |
| MN122161 | Netherlands | 2016 | Bird |
| MN122160 | Netherlands | 2016 | Bird |
| MN122159 | Netherlands | 2016 | Bird |
| MN122158 | Netherlands | 2016 | Bird |
| MN122157 | Netherlands | 2016 | Bird |
| MN122156 | Netherlands | 2016 | Bird |
| MN122155 | Netherlands | 2016 | Bird |
| MN122154 | Netherlands | 2016 | Bird |
| MN122153 | Netherlands | 2016 | Bird |
| MN122152 | Netherlands | 2016 | Bird |
| MN122151 | Netherlands | 2016 | Bird |
| MN122150 | Netherlands | 2016 | Bird |
| MN122149 | Netherlands | 2016 | Bird |
| MN122148 | Netherlands | 2016 | Bird |
| MN122147 | Netherlands | 2016 | Bird |
| MN122146 | Netherlands | 2016 | Bird |
| MN122145 | Netherlands | 2016 | Bird |
| MK796169 | Netherlands | 2016 | Bird |
| MK796168 | Netherlands | 2016 | Bird |
| MT188658 | Netherlands | 2016 | Bird |
| MN813490 | Netherlands | 2016 | Bird |
| MH891847 | Netherlands | 2016 | Bird |
| KY128482 | Netherlands | 2016 | Bird |
| KY426770 | Germany | 2016 | Bird |
| KY426769 | Germany | 2016 | Bird |
| KY426768 | Germany | 2016 | Bird |
| KY426767 | Germany | 2016 | Bird |
| KY426766 | Germany | 2016 | Bird |
| KY426765 | Germany | 2016 | Bird |
| KY426764 | Germany | 2016 | Bird |
| KY426763 | Germany | 2016 | Bird |
| KY426762 | Germany | 2016 | Bird |
| KY426761 | Germany | 2016 | Bird |
| KY426760 | Germany | 2016 | Bird |
| KY426759 | Germany | 2016 | Bird |
| KY426758 | Germany | 2016 | Bird |
| KY426757 | Germany | 2016 | Bird |
| KY426756 | Germany | 2016 | Bird |
| KY426755 | Germany | 2016 | Bird |
| KY426754 | Germany | 2015 | Bird |
| KY426753 | Germany | 2015 | Bird |
| KY426752 | Germany | 2015 | Bird |
| KY426751 | Germany | 2015 | Bird |
| KY426750 | Germany | 2015 | Bird |
| KY294723 | Germany | 2016 | Bird |
| KY294722 | Germany | 2016 | Bird |
| KY199558 | Germany | 2016 | Bird |
| KY199557 | Germany | 2016 | Bird |
| KY199556 | Germany | 2016 | Bird |
| KY114798 | Germany | 2016 | Bird |
| KY114797 | Germany | 2016 | Bird |
| LR989886 | Germany | 2018 | Bird |
| LR989887 | Germany | 2018 | Bird |
| LR989889 | Germany | 2019 | Bird |
| LR989890 | Germany | 2019 | Bird |
| LR989892 | Germany | 2019 | Bird |
| KU664609 | Germany | 2015 | Bird |
| KU664608 | Germany | 2015 | Bird |
| KM659877 | Germany | 2014 | Bird |
| KJ859683 | Germany | 2013 | Bat |
| KJ859682 | Germany | 2013 | Bat |
| KJ438781 | Germany | 2011 | Bird |
| KJ438780 | Germany | 2011 | Bird |
| KJ438779 | Germany | 2012 | Bird |
| KJ438778 | Germany | 2011 | Bird |
| KJ438777 | Germany | 2011 | Bird |
| KJ438776 | Germany | 2011 | Bird |
| KJ438775 | Germany | 2011 | Bird |
| KJ438774 | Germany | 2012 | Bird |
| KJ438773 | Germany | 2011 | Bird |
| KJ438772 | Germany | 2011 | Bird |
| KJ438771 | Germany | 2013 | Bird |
| KJ438770 | Germany | 2011 | Bird |
| KJ438769 | Germany | 2011 | Bird |
| KJ438768 | Germany | 2013 | Mosquito |
| KJ438767 | Germany | 2011 | Mosquito |
| KJ438766 | Germany | 2012 | Bird |
| KJ438765 | Germany | 2011 | Bird |
| KJ438764 | Germany | 2012 | Bird |
| KJ438763 | Germany | 2012 | Bird |
| KJ438762 | Germany | 2011 | Bird |
| KJ438761 | Germany | 2011 | Bird |
| KJ438760 | Germany | 2011 | Bird |
| KJ438759 | Germany | 2011 | Bird |
| KJ438758 | Germany | 2013 | Bird |
| KJ438757 | Germany | 2013 | Bird |
| KJ438756 | Germany | 2011 | Bird |
| KJ438755 | Germany | 2011 | Bird |
| KJ438754 | Germany | 2012 | Bird |
| KJ438753 | Germany | 2011 | Bird |
| KJ438752 | Germany | 2011 | Bird |
| KJ438751 | Germany | 2011 | Bird |
| KJ438750 | Germany | 2011 | Bird |
| KJ438749 | Germany | 2012 | Bird |
| KJ438748 | Germany | 2012 | Bird |
| KJ438747 | Germany | 2012 | Bird |
| KJ438746 | Germany | 2011 | Bird |
| KJ438745 | Germany | 2011 | Bird |
| KJ438744 | Germany | 2012 | Bird |
| KJ438743 | Germany | 2011 | Bird |
| KJ438742 | Germany | 2013 | Bird |
| KJ438741 | Germany | 2013 | Bird |
| KJ438740 | Germany | 2011 | Bird |
| KJ438739 | Germany | 2013 | Bird |
| KJ438738 | Germany | 2011 | Bird |
| KJ438737 | Germany | 2011 | Mosquito |
| KJ438736 | Germany | 2012 | Mosquito |
| KJ438735 | Germany | 2012 | Bird |
| KJ438734 | Germany | 2011 | Bird |
| KJ438733 | Germany | 2012 | Bird |
| KJ438732 | Germany | 2011 | Bird |
| KJ438731 | Germany | 2011 | Bird |
| KJ438730 | Germany | 2011 | Bird |
| KJ438729 | Germany | 2012 | Bird |
| KJ438728 | Germany | 2011 | Bird |
| KJ438727 | Germany | 2011 | Bird |
| KJ438726 | Germany | 2011 | Bird |
| KJ438725 | Germany | 2011 | Bird |
| KJ438724 | Germany | 2011 | Bird |
| KJ438723 | Germany | 2011 | Bird |
| KJ438722 | Germany | 2012 | Bird |
| KJ438721 | Germany | 2011 | Mosquito |
| KJ438720 | Germany | 2011 | Bird |
| KJ438719 | Germany | 2011 | Bird |
| KJ438718 | Germany | 2011 | Mosquito |
| KJ438717 | Germany | 2012 | Bird |
| KJ438716 | Germany | 2012 | Mosquito |
| KJ438715 | Germany | 2011 | Bird |
| KJ438714 | Germany | 2011 | Bird |
| KJ438713 | Germany | 2011 | Bird |
| KJ438712 | Germany | 2011 | Bird |
| KJ438711 | Germany | 2011 | Bird |
| KJ438710 | Germany | 2011 | Bird |
| KJ438709 | Germany | 2012 | Mosquito |
| KJ438708 | Germany | 2011 | Mosquito |
| KJ438707 | Germany | 2011 | Mosquito |
| KJ438706 | Germany | 2011 | Mosquito |
| KJ438705 | Germany | 2010 | Mosquito |
| HE599647 | Germany | 2011 | Bird |
| MH423836 | Germany | 2016 | Mosquito |
| LR989894 | Germany | 2019 | Bird |
| MT795154 | Germany | 2016 | Bird |
| MN419913 | Czech Republic | 2017 | Bird |
| MN419912 | Czech Republic | 2018 | Bird |
| MN419911 | Czech Republic | 2018 | Bird |
| MN419910 | Czech Republic | 2018 | Bird |
| MN419909 | Czech Republic | 2018 | Bird |
| MN419908 | Czech Republic | 2018 | Bird |
| MN419907 | Czech Republic | 2018 | Bird |
| MN419906 | Czech Republic | 2018 | Bird |
| MN419905 | Czech Republic | 2018 | Bird |
| MN419904 | Czech Republic | 2018 | Bird |
| MN419903 | Czech Republic | 2018 | Bird |
| MN419902 | Czech Republic | 2017 | Bird |
| MN419901 | Czech Republic | 2017 | Bird |
| MN419900 | Czech Republic | 2017 | Bird |
| MN419899 | Czech Republic | 2017 | Bird |
| MN419898 | Czech Republic | 2017 | Bird |
| MN419897 | Czech Republic | 2017 | Bird |
| MN419896 | Czech Republic | 2017 | Bird |
| MN419895 | Czech Republic | 2016 | Mosquito |
| MN395369 | Czech Republic | 2018 | Mosquito |
| MN384964 | Czech Republic | 2018 | Bird |
| MF063052 | Hungary | 2010 | Bird |
| MF063051 | Hungary | 2011 | Bird |
| MF063050 | Hungary | 2015 | Bird |
| MF063049 | Hungary | 2015 | Bird |
| MF063048 | Hungary | 2016 | Bird |
| MF063047 | Hungary | 2016 | Bird |
| MF063046 | Hungary | 2016 | Bird |
| MF063045 | Hungary | 2016 | Bird |
| MF063044 | Hungary | 2016 | Bird |
| MF063043 | Hungary | 2016 | Bird |
| MF063042 | Hungary | 2016 | Bird |
| EF206350 | Hungary | 2005 | Bird |
| MK230890 | Belgium | 2017 | Bird |
| MK230891 | Belgium | 2017 | Bird |
| MK230892 | Belgium | 2017 | Bird |
| MK230893 | Belgium | 2017 | Bird |
| KY315178 | Belgium | 2016 | Bird |
| KX977447 | Belgium | 2016 | Bird |
| KY263626 | Belgium | 2016 | Bird |
| KY263625 | Belgium | 2016 | Bird |
| KY263624 | Belgium | 2016 | Bird |
| MK419834 | Belgium | 2018 | Bird |
| MT863562 | France | 2018 | Bird |
| KY128481 | France | 2016 | Bird |
| KX601692 | France | 2015 | Bird |
| KX601691 | France | 2015 | Bird |
| KX601690 | France | 2015 | Bird |
| MG461313 | Israel | 2015 | Mosquito |
| MG461312 | Israel | 2004 | Mosquito |
| MG461311 | Israel | 2004 | Mosquito |
| MG461310 | Israel | 2004 | Mosquito |
| MG461309 | Israel | 2004 | Mosquito |
| MG461308 | Israel | 2015 | Mosquito |
| MG461307 | Israel | 2015 | Mosquito |
| MG461306 | Israel | 2015 | Mosquito |
| NC006551 | Austria | 2001 | Bird |
| MF991886 | Austria | 2017 | Human |
| JQ219843 | Austria | 2002 | Bird |
| AY453411 | Austria | 2001 | Bird |
| KU760915 | Spain | 2010 | Mosquito |
| MN813489 | Spain | 2009 | Mosquito |
| HQ833022 | Spain | 2009 | Mosquito |
| KF573410 | Spain | 2006 | Mosquito |
| MT891321 | Croatia | 2019 | Mosquito |
| MT891318 | Croatia | 2018 | Mosquito |
| MW803140 | Serbia | 2019 | Mosquito |
| MG888044 | Serbia | 2014 | Mosquito |
| MW001216 | Great Britain | 2020 | Bird |
| MT133690 | Sweden | 2019 | Bird |
| OU674388 | Luxembourg | 2020 | Bird |
| AF013412 | South Afica | 1959 | Mosquito |
| AY453412 | South Afica | 1959 | Mosquito |
| MF374485 | South Afica | 1959 | Human |
| MN813492 | South Afica | 1956 | Mosquito |
| MN813491 | Uganda | 2012 | Mosquito |
| MT241508 | Uganda | 2012 | Mosquito |
| MN813488 | Senegal | 2003 | Mosquito |
| MH727242 | Senegal | 2013 | Rat |
| MH727241 | Senegal | 2013 | Rat |
| MH727240 | Senegal | 2013 | Shrew |
| MH727239 | Senegal | 2013 | Rat |
| MH727238 | Senegal | 2013 | Rat |
| KC754957 | Senegal | 2007 | Mosquito |
| KC754956 | Senegal | 1993 | Mosquito |
| KC754954 | Senegal | 1974 | Mosquito |
| KC754958 | Central African Republic | 1969 | Mosquito |
| KC754955 | Central African Republic | 1981 | Human |

Table S1. List of sequences used in the present study
